# A Review of *Fibraurea tinctoria* and Its Component, Berberine, as an Antidiabetic and Antioxidant

**DOI:** 10.3390/molecules28031294

**Published:** 2023-01-29

**Authors:** Indah Purwaningsih, Iman Permana Maksum, Dadan Sumiarsa, Sriwidodo Sriwidodo

**Affiliations:** 1 Department of Chemistry, Faculty of Mathematics and Natural Science, Universitas Padjadjaran, Sumedang 45363, Indonesia; 2 Department of Medical Laboratory Technology, Poltekkes Kemenkes Pontianak, Pontianak 78124, Indonesia; 3 Department of Pharmaceutics and Pharmaceutical Technology, Faculty of Pharmacy, Universitas Padjadjaran, Sumedang 45363, Indonesia

**Keywords:** *Fibraurea tinctoria*, berberine, antidiabetes, antioxidant, in silico

## Abstract

Diabetes mellitus is a group of metabolic disorders characterized by hyperglycemia caused by resistance to insulin action, inadequate insulin secretion, or excessive glucagon production. Numerous studies have linked diabetes mellitus and oxidative stress. People with diabetes usually exhibit high oxidative stress due to persistent and chronic hyperglycemia, which impairs the activity of the antioxidant defense system and promotes the formation of free radicals. Recently, several studies have focused on exploring natural antioxidants to improve diabetes mellitus. *Fibraurea tinctoria* has long been known as the native Borneo used in traditional medicine to treat diabetes. Taxonomically, this plant is part of the Menispermaceae family, widely known for producing various alkaloids. Among them are protoberberine alkaloids such as berberine. Berberine is an isoquinoline alkaloid with many pharmacological activities. Berberine is receiving considerable interest because of its antidiabetic and antioxidant activities, which are based on many biochemical pathways. Therefore, this review explores the pharmacological effects of *Fibraurea tinctoria* and its active constituent, berberine, against oxidative stress and diabetes, emphasizing its mechanistic aspects. This review also summarizes the pharmacokinetics and toxicity of berberine and in silico studies of berberine in several diseases and its protein targets.

## 1. Introduction

Diabetes mellitus is a series of physiological dysfunctions characterized by hyperglycemia associated with abnormalities in carbohydrate, fat, and protein metabolism caused by resistance to insulin action, inadequate insulin secretion, or excessive glucagon production [1]. According to the *Diabetes Atlas 2021*, 537 million people are currently living with diabetes mellitus, which is expected to increase to 643 million by 2030. By 2045, this number will increase by 783 million, or nearly 46%. In addition, 541 million people are estimated to have impaired glucose tolerance by 2021, and more than 6.7 million people between the ages of 20 and 79 will die from diabetes-related causes [2].

Numerous studies have linked diabetes mellitus and oxidative stress [3,4]. Reactive oxygen species (ROS) are produced in cells in excess compared to the capacity of enzymatic and non-enzymatic antioxidants. This imbalance leads to oxidative stress, which damages membranes and essential macromolecules, including DNA, proteins, and lipids [3,5]. In diabetes mellitus, excess free radicals are formed through glucose oxidation and non-enzymatic protein glycation. Because of the cellular damage caused by the breakdown of the antioxidant defense system, diabetes-related problems such as nephropathy, retinopathy, and neuropathy can start to appear [6].

Currently, diabetes mellitus is considered a major global problem, and a truly successful treatment has yet to be found. Despite many current treatment options, hyperglycemia is often poorly controlled [7]. Agarwal et al. reported that the efficacy of antidiabetic drugs in achieving optimal glycemic control was only 41% of total patients [8]. Another study showed that although the use of antihyperglycemic drugs for treating diabetes mellitus has undergone many significant changes and developments, the number of patients who achieved the target HbA1c <7% decreased [9]. Data from the National Health and Nutrition Examination Survey (NHANES) showed that only 57% of people with diabetes achieved the target HbA1c <7% in 2003–2006, which fell to 52.5% in 2007–2010 [10] and fell again to 47.7% in 2011–2014 [11]. In addition, antidiabetic drugs still have limitations, such as disadvantages in the form of side effects and failure to change the course of diabetes complications [12]. For example, metformin, the most commonly prescribed biguanide oral hypoglycemic drug, causes gastrointestinal disturbances such as abdominal discomfort, diarrhea, anorexia, nausea, a metallic taste, and vitamin B_12_ deficiency [13,14]. Metformin is also linked to lactic acidosis, although the incidence is low [15,16,17].

Natural products are becoming more widely accepted and used as alternative therapies. Several studies have shown the increased use of natural products in patients with diabetes mellitus. This increase is due to the numerous side effects and high costs associated with antidiabetic drugs [18,19]. As targets for combination therapy, natural products have also been discovered to interact synergistically with antidiabetic drugs [20]. In addition, the combination with antidiabetic drugs allows lower drug doses and/or reduces the frequency of administration, thereby reducing side effects and increasing efficacy. Studies on natural products are still being conducted to evaluate their potential as antidiabetic agents and to identify the main structures that can be developed as new antidiabetic drugs [21].

There is a long history of using natural products and their development as antidiabetic drugs. More than 1200 medicinal plants have been tested for their antidiabetic potential, and 200 bioactive substances have been reported to have potent hypoglycemic effects [22]. Many of the currently used drugs are structurally derived from natural compounds. Some compounds with antidiabetic activity are galegine, phlorizine, and ruboxistaurine, obtained from plants; exenatide and trodusquemine, obtained from animals; and acarbose, miglitol, and voglibose, derived from microbes [23,24].

*Fibraurea tinctoria* is a yellow climbing plant known in Borneo as Akar Kuning (yellow root). This plant has long been known as the native Borneo in traditional medicine. The traditional medicinal uses of this plant include its leaves, roots, stems, and bark [25]. Ethnic groups in Kalimantan have traditionally used yellow root to treat malaria, jaundice, and diabetes [26]. In addition to *Fibraurea tinctoria*, there are two other plants, *Arcangelisia flava* (L) Merr and *Coscinium fenestratum* Colebr, also known as yellow root [27,28]. Taxonomically, these three plants are part of the Menispermaceae family [29]. The Menispermaceae family is widely known for producing a wide variety of alkaloids. Approximately 22 types of alkaloids have been identified in the Menispermacaceae family. Among them are protoberberine alkaloids, such as berberine [30].

Berberine is an isoquinoline alkaloid that has been used for diabetes treatment in traditional Chinese, Indian, and Middle-Eastern folk medicine for more than 400 years. Berberine is widely present in many traditional medicinal plants [31]. Berberine is an important natural alkaloid and is of great interest because of its wide and interesting spectrum of pharmacological activity. Berberine exhibited strong antihyperglycemic activity and positive effects in treating diabetic nephropathy, diabetic neuropathy, and diabetic cardiomyopathy [32]. In addition, several studies showed that berberine has antioxidant activity, which partially contributes to its efficacy against diabetes mellitus [33]. These observations make berberine a potentially promising drug for managing diabetes mellitus.

On this basis, this review focuses on the pharmacological activity of the *Fibraurea tinctoria* plant and its component, berberine, as an antidiabetic and antioxidant. This review will also discuss the pharmacokinetics and toxicity of berberine and in silico studies of berberine in several diseases and its protein targets.

## 2. *Fibraurea tinctoria*

*Fibraurea tinctoria* is a plant that produces color and is widely distributed in China, Indonesia, Malaysia, Thailand, Laos, Cambodia, and Vietnam [34]. This plant has a long history of use as a medicine in most countries in Asia [35]. In addition to treating dysentery, malaria, and gonorrhea, this plant is also used as an analgesic, antipyretic, antidote, and diuretic [25,34].

Su et al. isolated 45 compounds, including protoberberine alkaloids and furanoditerpenoids, from *Fibraurea tinctoria* stems. The furanoditerpenoid compounds isolated from this plant included epi-8-hydroxycolumbin (**1**), fibrauretin B (**2**), fibrauretin C (**3**), fibrauretin D (**4**), fibrauretin E (**5**), fibrauretin F (**6**), epi-12-palmatoside G (**7**), floribundic ester (**8**), fibraurin (**9**), fibraurinoside (**10**), fibleucin (**11**), fibleucinoside (**12**), chasmanthin (**13**), fibrauretin A (**14**), fibrauretinoside A (**15**), and epifibrauretinoside A (**16**). Other compounds that have been identified are palmatine (**17**), jatrorrhizine (**18**), columbamine (**19**), stepharanine (**20**), 8-trichloromethyl-7,8-dihydropalmatine (**21**), a mixture of *N*-(*p*-*trans*-coumaroyl)-tyramine (**22**) and *N*-(*p*-*cis*-coumaroyl)-tyramine (**23**), *N*-*trans*-feruloyl tyramine (**24**), *N*-*cis*-feruloyl tyramine (**25**), corydaldine (**26**), thalifoline (**27**), *p*-hydroxybenzaldehyde (**28**), vanillin (**29**), syrigaldehyde (**30**), methyl vanillate (**31**), methyl syringate (**32**), octadecyl (*E*)-ferulate (**33**), vanillic acid (**34**), syringic acid (**35**), ferulic acid (**36**), caffeic acid (**37**), fibraurecdyside A (**38**), makisterone A (**39**), a mixture of β-sitosterol (**40**) and sigmasterol (**41**), β-sitosterol 3-*O*-β-D-glucopyranoside (**42**), physcion (**43**), dehydrogormouregine (**44**), and thomasidioic acid dimethyl ester (**45**) [34].

Several studies have investigated the pharmacological activities of *Fibraurea tinctoria*. Nguyen-pouplin et al. conducted antimalarial and cytotoxicity tests on several plants grown in South Vietnam. *Fibraurea tinctoria* had the highest activity among the 38 plants examined for antimalarial activity, with IC_50_ values ranging from 0.40 to 1.10 μg/mL [27]. A study by Galappathie et al. on the antimicrobial and antifungal activities of several plants growing on the island of Borneo showed that the leaves and stems of *Fibraurea tinctoria* extracted using dichloromethane and methanol had activity against *Bacillus cereus* and *Staphylococcus aureus* with inhibition zones of 10 mm and 16 mm, respectively, and had a Minimum Inhibitory Concentration (MIC) and Minimum Bactericidal Concentration (MBC) values ranging between 25 and 100 μg/mL [25].

In other studies, floribundic ester and fibraurin from *Fibraurea tinctoria* showed significant anti-inflammatory activity when administered at 100 mg/kg to reduce carrageenan mouse paw edema. In addition, five components from *Fibraurea tinctoria,* namely, Epi-12-palmatoside G, fibraurin, fibraurinoside, fibrauretin A, and epifibrauretinoside A, inhibited NO production from macrophages at 1–4 μg/mL [34]. It was also found that the methanol extract of *Fibraurea tinctoria* inhibited cytochrome P450 3A4 with an IC_50_ of 5.10 μg/mL [36].

Keawpradub et al. measured the antioxidant activity, acute toxicity, and cytotoxicity of *Fibraurea tinctoria* grown in Thailand. Antioxidant testing using the DPPH method (1,1-diphenyl-2-picrylhydrazil) showed that the chloroform and methanol extracts of *Fibraurea tinctoria* exhibited potent antioxidant activity with IC_50_ values of 78.80 and 83.60 μg/mL. In contrast, petroleum ether and water extracts showed weak antioxidant activity with IC_50_ > 100 μg/mL. The chloroform extracts of *Fibraurea tinctoria* showed cytotoxic activity against brine shrimp and the human cancer cell line MCF-7 with LC_50_ and IC_50_ values of 252.70 μg/mL and 11.20 μg/mL, respectively. In contrast, petroleum ether, methanol, and water extracts had LC_50_ values of >1000 μg/mL and IC_50_ values of >50 μg/mL [37]. *Fibraurea tinctoria* also showed moderate antiproliferative activity against human cervical cancer (HeLa), oral cancer (KB), and human hepatoma cancer (HepG2), with GI_50_ values of 39.61 μg/mL, 31.50 μg/mL, and 89.11 μg/mL, respectively [38,39].

## 3. Berberine

Berberine (18, 5,6-dihydro-9,10-dimethoxybenzo(g)-1,3-benzodioxolo (5,6-a) quino-lizinium) is a benzyl tetra isoquinoline alkaloid that naturally occurs in the plant families Ranunculaceae, Rutaceae, Berberidaceae, Annonaceae, Papaveraceae, and Menispermaceae [30,40,41] and has been widely used in traditional medicine for conditions such as dysentery [42], diarrhea [43,44,45,46,47,48], stomatitis [49], and hepatitis [50,51].

**Figure 1:**
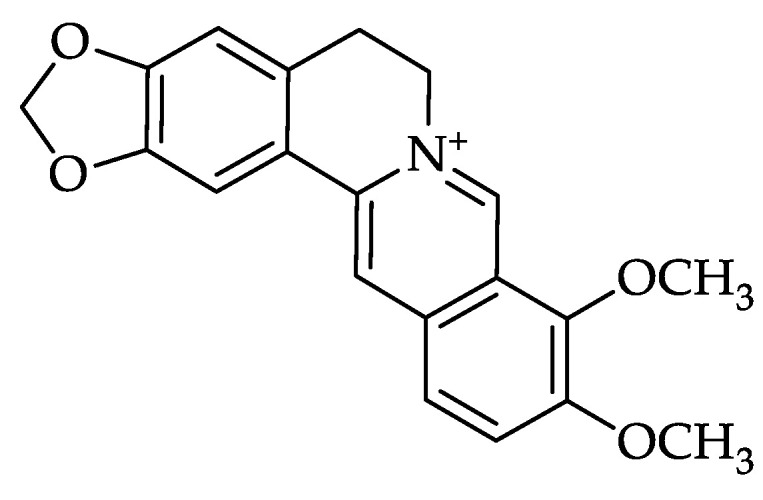
Molecular structure of berberine.

Several studies reported that berberine has many pharmacological activities, including antihyperlipidemic, antidiabetic, antiobesity, anticancer, anti-inflammatory, antioxidant, neuroprotective, cardioprotective, immunomodulatory, and other effects [52,53,54,55]. Recently, many studies have been conducted to examine the role of berberine in COVID-19 [56,57,58,59] (Figure 1).

### 3.1. Pharmacokinetics of Berberine

Clinically, berberine is administered orally. However, pharmacokinetic studies have shown that berberine has low bioavailability (<5%) [60]. Berberine has an extremely low absolute bioavailability, possibly because of its nature as a quaternary ammonium base. The quaternary ammonium group in its structure exhibits strong hydrophilicity and poor permeability through the cell membrane. Low drug bioavailability results from the restriction of transmembrane transport and intestinal absorption [61].

In addition to being caused by its structure, the low bioavailability of berberine after oral administration is caused by its low permeability, efflux mediated by P-glycoprotein (P-gp-), hepatobiliary excretion, and self-aggregation. The low bioavailability of berberine is also associated with its poor absorption and rapid metabolism in the body (liver and intestine) [62]. As a result of this metabolism, the body produces berberine metabolites known to contribute significantly to the pharmacological effects of berberine [40]. Berberine is also known to have a pKa value = 15, which causes berberine to be in an ionized form in the physiologic condition, so the amount of berberine that can be absorbed after oral administration is low [63].

After oral administration, only 0.50% of the berberine dose can enter the blood circulation, causing low concentrations of berberine in the plasma. Approximately 56% of orally administered berberine is not absorbed owing to P-glycoprotein-mediated efflux and self-aggregation, and 43.50% is metabolized by enterocytes [62]. Liu et al. reported that 99.50% of the oral dose of berberine disappeared during first-pass metabolism in the gastrointestinal tract [64].

The interference of P-glycoprotein with berberine absorption is one of the reasons for its low bioavailability. P-glycoprotein is a vital transporter protein found in epithelial cell membranes that functions as an efflux pump that actively removes berberine from luminal mucosal cells. Therefore, one of the strategies used to increase the bioavailability of berberine is the concurrent administration of berberine with a P-glycoprotein inhibitor. These inhibitors can reduce the efflux of berberine and increase its absorption in the intestine, thereby affecting its pharmacokinetic profile [65]. Some examples of P-glycoprotein inhibitors include tetrandrine [66], D-α-tocopheryl polyethylene glycol 1000 succinate (TPGS) [67], glycine (GLY) [68], cyclosporine A (CsA) [69], and oligomeric proanthocyanidins (OPCs) [70].

The gut microbiota plays a vital role in the absorption process. Nitroreductase (NR) from the gut microbiota can convert berberine into a more easily absorbed form, dihydroberberine. Dihydroberberine is absorbed in the intestine 5–10 times more effectively than berberine [71]. However, dihydroberberine is unstable and can be quickly converted back into berberine in the intestinal wall through non-enzymatic oxidation processes and then enters the bloodstream. Therefore, nitroreductase (NR) from the gut microbiota is vital for regulating berberine blood levels [72].

After entering the blood, berberine is distributed mainly to tissues, causing low levels of berberine in the plasma. Berberine is widely distributed in the liver, kidneys, muscles, lungs, brain, heart, pancreas, and fat tissues. Berberine is predominantly found in the liver [73]. The pharmacokinetic profile showed that four hours after administration, the tissue concentration of berberine was 70 times higher than the plasma concentration. In addition, berberine is known to be relatively stable in tissues (e.g., liver, muscle, brain, heart, and pancreas) [64].

Besides absorption and distribution factors, metabolic factors also contribute to the low levels of berberine in the plasma. After oral administration, berberine undergoes several metabolic pathways, resulting in extremely low plasma berberine levels. The major pathways of berberine metabolism are demethylation, demethylenation, reduction, hydroxylation (phase I metabolism), and subsequent glucuronidation, sulfation, and methylation (phase II metabolism) [74,75,76]. Berberine is metabolized mainly by the liver and intestinal flora, and this metabolic process causes the content and structure of berberine to change, which in turn affects its pharmacological activity [77].

Several studies have been conducted on the metabolic processes of berberine and its metabolites. Pan et al. found three metabolites in human urine samples after the administration of berberine chloride, identified as jatrorrhizine 3-sulfate, demethyleneberberine 2-sulfate, and thalifendine 10-sulfate, where the main metabolite is demethyleneberberine 2-sulfate [78]. Tsai et al. reported that berberine is metabolized in the rat liver by phase I (demethylation) and phase II (glucuronidation), which was concluded based on ion peaks at *m*/*z* 322 and 498 in bile samples determined using liquid chromatography/mass spectrometry (LC/MS) [78].

In another study in rats, berberine was metabolized in the liver by cytochrome P450 enzymes, including phase I (oxidative demethylation), followed by phase II (glucuronidation). In phase I metabolism, several metabolites, such as berberrubine, thalifendine, demethyleneberberine, and jatrorrhizine, were found, and their respective glucuronide conjugates were found in most tissues, where berberrubine was the main metabolite in plasma [63,74,79].

Research conducted by Xu et al. found 16 berberine metabolites, namely, 10 metabolites resulting from phase I and 6 metabolites from phase II metabolism. These metabolites have been identified in the plasma, feces, bile, and other tissues [80]. Among these metabolites, berberrubine, thalifendine, demethyleneberberine, and jatrorrhizine have relatively high plasma levels [65]. Similar findings were also reported by Wang et al., who identified more than twenty berberine metabolites in humans and mice [40] (Table 1).

Wang et al. identified berberine metabolites in plasma, urine, bile, and feces after the administration of 100 mg/kg/day berberine for three days in rats using ultra-high-performance liquid chromatography/quadrupole time-of-flight mass spectrometry (UHPLC/Q-TOF-MS). This study identified 97 metabolites, including 68 in urine, 45 in plasma, 44 in bile, and 41 in feces, where demethylation, demethylenation, reduction, hydroxylation, and subsequent glucuronidation, sulfation, and methylation are the major metabolic pathways of berberine in vivo [76]. In plasma, the phase I metabolites found were berberrubine, demethyleneberberine, and jatrorrhizine, where berberrubine was found at higher levels than demethyleneberberine and jatrorrhizine, whereas jatrorrhizine-3-*O*-β-D-glucuronide, jatrorrhizine-3-*O*-sulfate, thalifendine-10-*O*-β-D-glucuronide, berberrubine-9-*O*-β-D-glucuronide, demethyleneberberine-2-*O*-sulfate, and demethyleneberberine-2-*O*-β-D-glucuronide were phase II metabolites found at high levels in plasma [81].

Berberine is excreted via the hepatobiliary system and kidneys [72]. However, most berberine is excreted through the kidneys (urine) and feces. In some animals, berberine is excreted in the bile, and this excretion pathway occurs very slowly due to hepatoenteric circulation. Studies have shown that in some research subjects, both in test animals and in humans, berberine administered through different routes, such as oral or intravenous administration, will cause different excretion processes [77]. Ma et al. reported that the excretion process of berberine after the oral administration of 200 mg/kg in rats was mostly through feces (22.70%), followed by excretion in lesser amounts through urine (0.09%) and bile (9.20 × 10^−6^%) in the form of berberine (19.07%) and its metabolites (3.76%) [82]. A similar study by Feng et al. showed that berberine is excreted mainly through feces after oral administration in rats. Berberine is difficult to absorb in the intestine because it is in an ionized form under physiological conditions, so it will be excreted through the feces as its parent component (unabsorbed berberine). Berberine is excreted through feces in the form of berberrubine (18.60%) and bile in the form of berberrubine (2.62%) and berberrubine-9-*O*-β-D-glucuronide (0.36%). A large amount of excreted berberrubine indicates that berberine undergoes a metabolic process to become berberrubine with the help of metabolizing enzymes in the intestinal wall or intestinal microbes [81]. Studies in humans showed that after berberine administration (0.20 g/day), only approximately 0.01% of berberine is eliminated directly through the urine [83].

**Figure 2:**
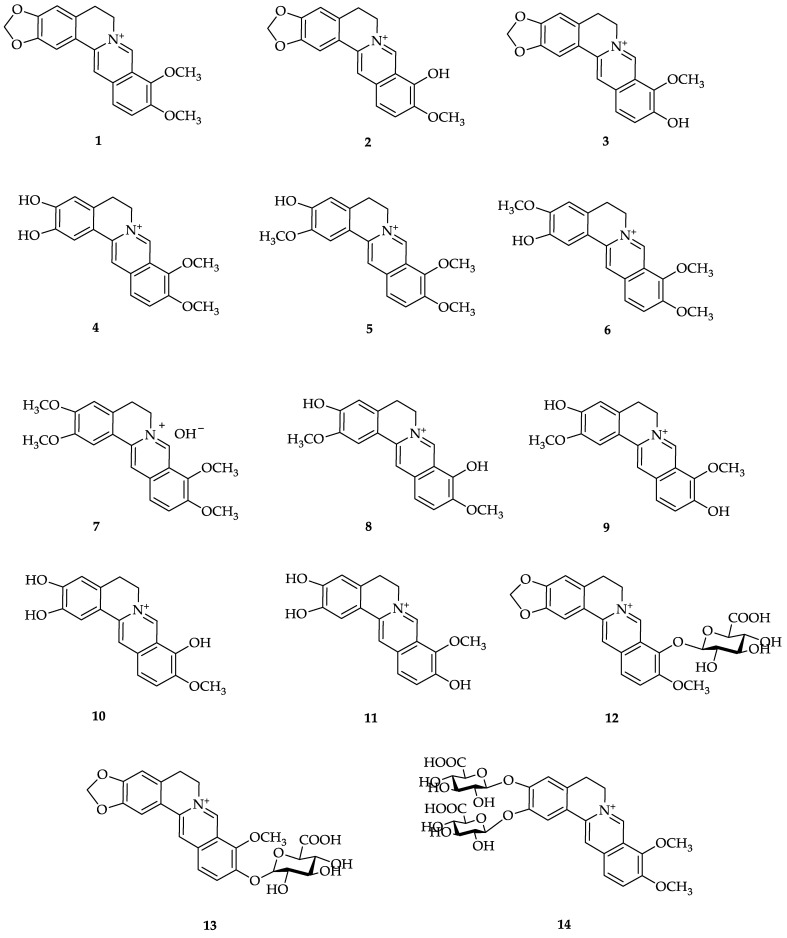

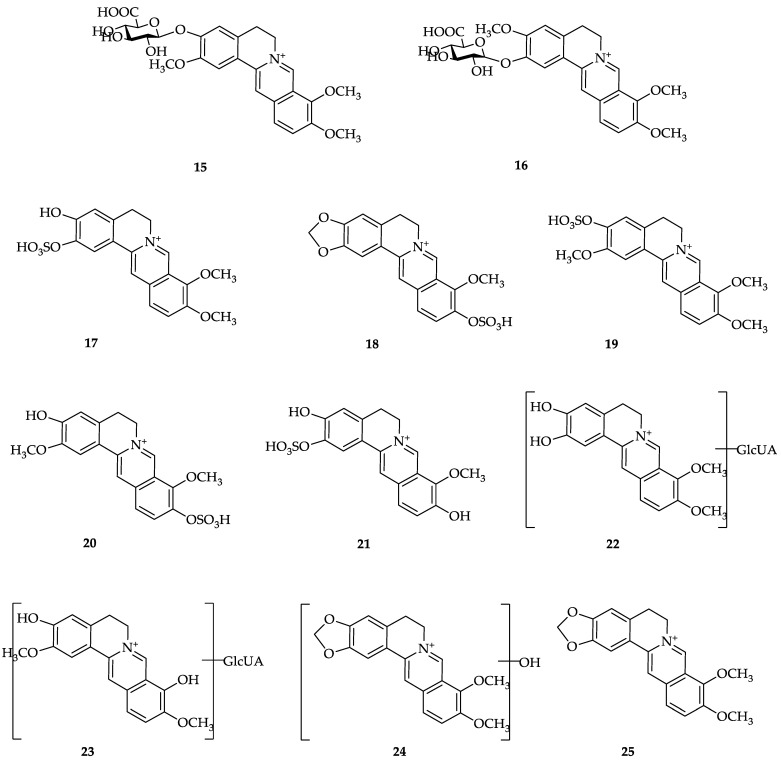
Structures of berberine and its metabolites. (**1**) Berberine; (**2**) berberrubine; (**3**) thalifendine; (**4**) demethyleneberberine; (**5**) jathorrhizine; (**6**) columbamine; (**7**) palmatine; (**8**) 3,9-demethylpalmatine; (**9**) 3,10-demethylpalmatine; (**10**) 2,3,9-trihydroxiberberine; (**11**) 2,3,10-trihydroxiberberine; (**12**) berberrubine-9-*O*-β-D-glucuronide; (**13**) thalifendine-10-*O*-β-D-glucuronide; (**14**) demethyleneberberine-2,3-di-*O*-β-D-glucuronide; (**15**) jatrorrhizine-3-*O*-β-D-glucuronide; (**16**) columbamine-3-*O*-β-D-glucuronide; (**17**) demethyleneberberine-2-*O*-sulfate; (**18**) thalifendine-10-*O*-sulfate; (**19**) jatrorrhizine-3-*O*-sulfate; (**20**) 3,10-demethylpalmatine-10-*O*-sulfate; (**21**) 2,3,10-trihydroxyberberine-2-*O*-sulfate; (**22**) glucuronide of demethyleneberberine; (**23**) glucuronide of 3,9-demethylpalmatine; (**24**) hydroxylated berberine; (**25**) dihydroberberine [40].

The three studies above showed that most berberine is excreted through feces, followed by urine and bile in smaller amounts (feces > urine > bile). However, research conducted by Guo et al. in mice administered berberine at 10 mg/kg intraperitoneally (i.p.) showed that berberine is found at high levels in urine, where the amount of berberine excreted in urine is approximately two times higher than that in feces. In addition, 11 berberine metabolites were found, including 5 in feces and 6 in urine [79] (Figure 2).

### 3.2. Berberine Toxicity

Kheir et al. conducted a study on the LD_50_ value of berberine administered in mice using three different routes of administration: intravenous injection (i. v.), intraperitoneal injection (i. p.), and oral administration via intragastric injection (i. g.). Berberine is known to have an LD_50_ value of 9.04 ± 0.80 mg/kg when administered by intravenous injection and 57.61 ± 23.32 mg/kg by intraperitoneal injection. The safe dose of berberine in mice when administered orally was 20.80 g/kg body weight. It is known that mice have a metabolic rate approximately seven times higher than that of humans, so the safe dose of berberine in humans is 1/7 of 20.81 g/kg, which is 2.97 g/kg body weight. The highest bioavailability of berberine was by intravenous injection, followed by intraperitoneal and intragastric injection (i.v > i.p > i.g). Bioavailability is known to affect LD_50_ values. Variations in the concentration of berberine in the blood cause different toxicity levels; the greater the bioavailability of berberine, the lower the LD_50_ value (more toxic) [84].

Another study found that in mice, the LD_50_ values of berberine upon intraperitoneal and oral administration were 23 and 329 mg/kg, respectively [85]. However, Anis et al. found that the LD_50_ value of berberine administered via the intraperitoneal injection was 50 mg/kg body weight [86].

Research by Lan et al. on the effects and safety of berberine in patients with type 2 diabetes mellitus, hyperlipidemia, and hypertension showed that the incidence of toxic side effects is strongly correlated with the dose of berberine administered. The higher the dose of berberine, the higher the risk of toxic side effects. A meta-analysis of 27 studies revealed that berberine could cause a few side effects; however, the frequency of these side effects is low, and major side effects during therapy do not harm the body’s vital organs. Berberine can be utilized as an alternative therapy for patients with poor socioeconomic status because it is generally safe for treating diabetes, hyperlipidemia, and hypertension [87].

Kidneys are the primary excretory organs and play an important role in the body. Kidneys mediate the toxicity of various drugs, environmental pollutants, and natural substances that enter the body. Disturbances in kidney function can affect the toxicity of a compound and cause changes in biochemical parameters related to kidney function. Several drugs, environmental pollutants, and natural substances can cause kidney damage, commonly referred to as nephrotoxicity [88,89]. Several studies on the effect of berberine on the kidneys have been conducted. The results showed that berberine had a nephroprotective effect against kidney damage induced by several compounds, including cisplatin, lead, methotrexate, doxorubicin, diclofenac, and gentamicin. The nephroprotective effect of berberine is mediated through many mechanisms, including the inhibition of oxidative/nitrosative stress, inflammation, autophagy, apoptosis, mitochondrial dysfunction, and fibrosis [90,91,92,93,94,95,96,97]. Berberine inhibits oxidative stress, inflammation, apoptosis, and autophagy in the kidney by activating Nrf2/HO-1 and inhibiting the expression of JNK/p38MAPKs/PARP/Beclin-1 [98].

Berberine protects the liver and kidneys from toxicity induced by ferrous sulfate by decreasing lipid peroxidation by scavenging free radicals and by chelating iron, thereby reducing the concentration of iron, which acts as a catalyst for lipid peroxidation [99]. Berberine is also known to protect against mercury-induced liver and kidney damage by increasing the expression of Bcl-2 protein in the liver and kidneys [100].

The most common mRNA modification is N6-methyladenosine (m6A). m6A participates in various physiological and pathological processes, such as metabolism, inflammation, and apoptosis. m6A plays an important role in cisplatin-induced acute renal failure, and berberine can alleviate this process [101]. In addition, berberine relieves cisplatin-induced acute renal failure by regulating mitophagy via the PINK 1/Parkin pathway in renal tubular epithelial cells (RTECs) [102].

Renal tubular epithelial-to-mesenchymal transition (EMT) and renal tubular interstitial fibrosis are the main pathological changes in diabetic nephropathy that cause kidney disease in the final stage. Berberine exerts a therapeutic effect on diabetic nephropathy by inhibiting EMT in the renal tubules and interstitial fibrosis in the kidney. The berberine-mediated inhibition of EMT occurs via the regulation of the Notch/Snail pathway [103]. Berberine can potentially prevent or treat tubulointerstitial fibrosis in diabetic nephropathy by activating the Nrf2 pathway and inhibiting TGF-β/Smad/EMT signaling [104].

Pentoxifylline is a xanthine-derived drug used to treat peripheral arterial disease and nephropathy in patients with diabetes. Al-Kuraishy et al. found that the combined administration of berberine and pentoxifylline provided a significantly more renoprotective effect than either berberine or pentoxifylline alone in diclofenac-induced acute renal failure [105].

Berberine has been reported to reduce the levels of uric acid (UA), blood urea nitrogen (BUN), creatinine (CRE), and pro-inflammatory cytokines (IL-1β and IL-18) in both the serum and kidneys. High uric acid levels are known to cause severe inflammatory effects that can progress to kidney organ dysfunction and even cause kidney damage. Berberine is known to exert nephroprotective effects by inhibiting the expression of URAT1 and suppressing the activation of the NLRP3 inflammasome, which can prevent inflammation in the kidney [106]. Another study showed that berberine has a protective effect against renal fibrosis caused by diabetic nephropathy by decreasing the expression of transforming growth factor (TGF-β) and α-smooth muscle actin (α-SMA) in the kidneys. Therefore, berberine can be used to treat diabetic nephropathy [107].

Several studies on berberine in plants and its effects on the kidneys have also been conducted. Studies have shown that *Coptis chinensis* has hepatoprotective and nephroprotective effects against cinnabar (HgS)-induced kidney damage [108]. Another study reported that *Berberis baluchistanica* (Berberidaceae) has nephroprotective effects against gentamicin-induced kidney damage [109], and *Berberis vulgaris* L (Berberidaceae) has nephroprotective effects against lead acetate-induced kidney damage through its antioxidant and metal-chelating abilities [110].

### 3.3. In Silico Studies of Berberine

Finding and developing drugs is important because it takes a long time, and it is costly to produce a potential new drug. The new drug discovery process begins with identifying target proteins, validating target proteins, identifying lead compounds, and optimizing lead compounds. In addition, before a new drug is released to the market for sale, it must first go through pre-clinical and clinical trials to obtain a distribution permit. Identifying the target protein takes the longest of these stages [111].

The search for target proteins and lead compounds is an important stage in the early research involving drug search processes. The search for target proteins aims to identify and validate suitable target proteins for disease therapies. Target proteins include receptors, enzymes, transporters, ion channels, transcription factors, and other therapeutic targets [112]. Conversely, the search for lead compounds aims to identify new molecules and compounds that act on target proteins. The target protein validation stage usually requires in vitro and in vivo tests using animal models, which can proceed to human testing after passing these two tests [113].

In silico is a term that means performed on a computer or through computer simulation. In silico drug design is a form of computer-based modeling that applies technology to the drug discovery process [111]. In silico methods have been widely developed and used extensively in pharmacology to search for new drugs because they significantly contribute to initial drug research, especially in identifying target proteins and lead compounds [112]. This method can predict the affinity between drug molecules and target proteins, explaining a drug molecule’s absorption, distribution, metabolism, excretion, and toxicity (ADMET) and its physicochemical characteristics [114].

The in silico method can reduce the number of test animals used in pharmacological tests. In addition, this method assists in the rational and safe design of new drug candidates and supports chemists and pharmacologists during the drug search process. It would be impossible to test millions of molecules in a lab against a given target, but the computerized screening process helps researchers choose a small set of molecules that can then be tested in a wet lab. Therefore, the in silico approach provides a great opportunity to identify target proteins quickly and accurately, thereby saving time and money in discovering new drugs [115] (Table 2).

## 4. Antioxidant Activity of Berberine

Oxidative stress in experimental diabetic animal models was first observed in 1982. Studies have shown that oxidative stress plays an important role in all cases of diabetes mellitus, especially type 2 diabetes mellitus and the pathogenesis of diabetes complications [169]. Oxidative stress can occur because of glucose and lipid metabolism abnormalities, leading to hyperglycemia and dyslipidemia. Hyperglycemic conditions are known to worsen and exacerbate the formation of ROS and constitute a significant factor in oxidative stress and glucotoxicity in pancreatic beta cells [170].

Diabetes causes severe metabolic imbalances and non-physiological changes in many tissues, especially in the pancreas. Markers of oxidative stress are elevated in the pancreatic islets of Langerhans in diabetic rats, and oxidative stress is related to impaired insulin secretion and insulin action [3,171]. Another study showed that ROS are strongly associated with the incidence of insulin resistance, resulting in pancreatic beta-cell damage and apoptosis in type 2 diabetes mellitus [31]. ROS are also known to increase the expression of tumor necrosis factor-α (TNF-α), which causes oxidative stress. Increased TNF secretion is associated with insulin resistance caused by obesity, a risk factor for type 2 diabetes mellitus [4].

The increased number of ROS can also act as second messengers and regulate the function of several proteins, including IκB kinase (IKKβ), protein kinase C (PKC), and Kelch-like ECH-associated protein 1 (Keap1), through interactions with cysteine residues (known as a “redox sensor”) of this protein. Redox modification is the term for modulating intracellular redox sensors by ROS. These protein redox changes activate different downstream signaling pathways that play important roles in impaired insulin secretion and insulin resistance, critical for the onset of diabetes mellitus and its consequences [172,173].

Since oxidative stress and diabetes are closely related, various initiatives to promote the health of diabetic patients have been carried out, one of which is to provide dietary supplements containing antioxidants [174]. Berberine is an isoquinoline alkaloid with antioxidant activity and can inhibit the formation of free radicals (ROS) and increase the activity of antioxidant enzymes. In addition, berberine improves mitochondrial function by decreasing oxygen consumption and increasing the mitochondrial membrane potential to prevent oxidative damage [175].

Several mechanisms by which berberine reduces oxidative stress in diabetes mellitus have been reported.

### 4.1. Berberine Scavenges ROS and Blocks ROS Generation

Shirwaikar et al. investigated the antioxidant activity of berberine in vitro. Berberine has potential as an antioxidant because it can capture superoxide free radicals directly in systems containing alkaline dimethyl sulfoxide (DMSO). Other antioxidant mechanisms of berberine include removing oxygen, capturing ROS and RNS or their precursors, inhibiting the formation of ROS and RNS, binding metal ions needed as catalysts for ROS formation, and regulating endogenous antioxidants [170]. Similar results were shown in a study by Jung et al., where berberine has ONOO^–^ scavenging activity [176]. However, a study by Choi et al. showed that berberine could not stabilize hydroxyl radicals but showed a strong ability to stabilize superoxide anions [177].

### 4.2. Berberine Chelates Metal Ions

Several metals, such as iron (Fe), copper (Cu), and calcium (Ca), are involved in the oxidation process. Fe plays a role in lipid peroxidation reactions and produces free radicals [178]. The ability of berberine to chelate Fe^2+^ is still debated. Studies conducted by Shiwarkal et al. demonstrated that berberine shows metal-chelating activity [170], whereas other studies showed otherwise [179,180]. An in vivo study on iron-overloaded mice by Aalikhani et al. showed that berberine decreased total iron levels in the liver, kidney, and lung tissues. Berberine prevents lipid peroxidation by reducing excess iron and chelating it in tissues, which is equivalent to DFO, an iron-chelating drug [181].

### 4.3. Berberine Regulates Enzyme Activity

Free radicals are physiologically active biomolecules produced by metabolic pathways and immune cells. Free radicals can be derived from molecular oxygen derivatives such as reactive oxygen species (ROS) or molecular nitrogen derivatives such as reactive nitrogen species (RNS). The enzymes responsible for forming free radicals are the mitochondrial enzymes NADPH oxidase (NOX), xanthine oxidase (XO), cyclooxygenase (COX), lipoxygenase (LOX), nitric oxide synthases (NOS), monoamine oxidase (MAO), myeloperoxidase (MPO), and cytochrome P450 enzymes. The body requires specific quantities of free radicals to regulate several cell physiological activities. However, when the number of free radicals increases above the physiological range, it will cause a condition called oxidative stress [182]. Most cells have an intrinsic defense mechanism involving various enzymes, such as superoxide dismutase (SOD), catalase (CAT), glutathione peroxidase (GPx), GSH reductase (GR), and glutathione transferase (GST), which can protect cells from free radical attack [183].

#### 4.3.1. Berberine Enhances Antioxidant Enzyme Activity and Inhibits Lipid/Protein Peroxidation

In the body, free radicals are converted to hydrogen peroxide by superoxide dismutase (SOD). Hydrogen peroxide is converted into hydroxyl radicals (*OH) during the propagation stage. These hydroxyl radicals cause lipid peroxidation in cell membranes, resulting in cell damage. If allowed to continue, this situation causes an imbalance between free radicals and endogenous antioxidants, known as oxidative stress. Therefore, one of the oxidative stress markers is increased malondialdehyde (MDA), a lipid peroxidation product formed under oxidative stress conditions [184]. Hydrogen peroxide can also be converted into oxygen and water by catalase (CAT) or glutathione peroxidase (GPx). Glutathione (GSH) is a substrate required for the antioxidant activity of GPx. When catalyzing this process, the disulfide bonds of GSH bind to form oxidized glutathione (GSSG), and glutathione reductase (GR) can recycle GSSG into GSH again by oxidizing NADPH. When cells are subjected to oxidative stress, GSSG accumulation occurs, and the GSH/GSSG ratio decreases [185].

GST is generally considered a phase 2 enzyme, mainly in electrophilic compounds for detoxification. Additionally, numerous investigations have demonstrated that GST can catalyze the breakdown of lipid hydrogen peroxide created by oxidative damage to lipid molecules [186].

Protein carbonyl is a biomarker of protein oxidation. Protein carbonylation is an irreversible oxidative protein modification that is an early marker of oxidative-stress-related disorders. Protein carbonyl levels are elevated in many tissues because of acute or prolonged oxidative stress. Protein carbonyl levels are significantly increased in type 2 diabetes [187].

It is known that in diabetes mellitus, there is an increase in GPx mRNA expression and, conversely, a decrease in SOD levels caused by a reduction in SOD mRNA expression. Berberine was reported to restore the GPx levels and SOD mRNA expression in type 2 diabetic animal models back to normal conditions, whereas this ability was not shown by metformin or glibenclamide [188,189].

Several studies related to the antioxidant activity of berberine, both in vitro using cell cultures grown on media containing high glucose and in vivo in experimental animals with streptozotocin- or alloxan-induced diabetes mellitus, have been carried out. Berberine reduces lipid/protein peroxidation biomarkers and increases antioxidant enzyme activity, which counteracts free radicals and overcomes oxidative stress (Table 3).

#### 4.3.2. Berberine Inhibits the Production of Oxidase

The NADPH oxidase (NOX) family is a major source of ROS in eukaryotic cells. NOX consists of several isoforms associated with electron transfer and is involved in many essential human physiological functions [209]. NOX activation is related to the onset of diabetes [210]. NOX is a potential target for treating diabetes mellitus and its complications [211].

Berberine can reduce oxidative stress by decreasing the expression of NOX. Berberine suppresses NOX4 overexpression and decreases ROS production in macrophages, endothelial cells, and the vascular endothelium [212,213,214]. Another study showed that NOX2 and NOX4 were detected in the aorta of streptozotocin-induced apolipoprotein E-deficient (apoE^−/−^) in type 1 diabetic mice [215]. NOX1 and NOX4 were overexpressed in diabetic rats (db/db) [216]. NOX4 is the main NOX isoform in human endothelial cells. The overexpression of NOX4 activates ROS in human endothelial cells [217]. NOX4 is also the primary source of ROS formation in blood vessel walls [218,219].

NOX is negatively regulated by AMPK activation [220,221]. AMPK plays an important role in the antioxidant [188,189] and antidiabetic activity of berberine [222]. In diabetes mellitus, AMPK becomes inactive [223]. Studies have shown that oxidative stress can regulate AMPK activity [224]. AMPKα1 is more sensitive to oxidative stress [225], while AMPKα2 is more sensitive to increased AMPK concentrations [225,226]. Berberine is an AMP-activated protein kinase (AMPK) activator [222]. AMPK activation causes the downregulation of NOX, inhibits ROS formation, and increases SOD expression [227].

It has been demonstrated that XO is a significant source of H_2_O_2_ and free radical production in cells and tissues. XO produces peroxide free radicals by catalyzing the oxidation of hypoxanthine and xanthine. Several studies have shown that XO activity is associated with diabetes mellitus [228,229]. Berberine showed the inhibition of xanthine oxidase with an IC_50_ of 23.64 μM [230].

Nitric oxide (NO) is a free radical produced by nitric oxide synthase (NOS) and is a biomarker of oxidative stress. NOS consists of three isoforms: neuronal (nNOS), inducible (iNOS), and endothelial (eNOS). Studies have shown an increase in NO levels in patients with diabetes mellitus [231]. Berberine has been reported to reduce NO levels [196,205]. Under hyperglycemic conditions, NO levels increase through the upregulation of iNOS. In vivo studies have shown that berberine reduced iNOS levels in diabetic rats [203,204]. In another study, berberine reduced monoamine oxidase (MAO) activity in STZ-induced diabetic rats [208]. Recently, MAO has been identified as a source of reactive oxygen species (ROS) that cause oxidative stress in diabetes mellitus [232].

### 4.4. Berberine Effects on Signal Transduction Pathway and Cell Antioxidant Response

Nuclear factor erythroid 2-related factor 2 (Nrf2) is a transcription factor that activates antioxidant enzymes to protect cells from damage induced by oxidative stress. Nrf2 deficiency increased ROS production [233]. Berberine is an Nrf2 activator. Berberine protects against oxidative stress by increasing Nrf2 and Hmox-1 expression [234]. Hsu et al. showed that berberine acts against oxidative stress by activating the PI3K/Akt/Nrf2-dependent pathway [235]. In addition, a further study indicated that berberine enhanced nuclear Nrf2 translocation and HO-1 expression [236]. Another study reported that berberine increased the transcription of Nrf2-targeted antioxidative genes (NADPH quinone oxidoreductase-1 (NQO-1) and heme oxygenase-1 (HO-1)), as well as the nuclear localization and phosphorylation of the Nrf2 protein. The Nrf2-mediated activity of berberine depends on AMPK activation [237]. Deng et al. investigated the effects of berberine in rats with NAFLD. Berberine alleviates hepatic oxidative stress, which may be partly attributed to the activation of the Nrf2/ARE signaling pathway [238].

A study by Zhu et al. showed that berberine protects the liver from oxidative stress by increasing sirtuin 1 (SIRT1) levels [239]. SIRT1 is a class III histone deacetylase belonging to the sirtuin family. It regulates several important processes, such as metabolism, oxidative stress, aging, and apoptosis, through the deacetylation of various substrates. SIRT1 inhibition increases ROS levels, and conversely, oxidative stress conditions such as excessive ROS can reduce SIRT1 expression/activity [240]. SIRT1 stimulates antioxidant expression, repairs ROS-induced cell damage, and prevents cell dysfunction [241]. SIRT1 is currently receiving much attention because it fights oxidative stress through several mechanisms, such as the Sirt1/FOXOs, Sirt1/NF-κB, Sirt1/NOX, Sirt1/SOD, and Sirt1/eNOs pathways [242,243]. SIRT1 also protects cells from oxidative stress by increasing catalase activity and blocking p53-induced apoptosis through p53 deacetylation and manganese SOD (MnSOD) induction [244]. Recent studies have shown that SIRT1 can fight oxidative stress in diabetes mellitus by mediating important signals, such as AMPK, NADPH oxidase, endothelial NO synthase, mTOR, and miRNAs [245].

Berberine increases the expression of UCP2 (uncoupling protein 2) [246]. UCP2 is an important member of the uncoupling protein (UCP) family, mitochondrial transport proteins located in the inner mitochondrial membrane, constituting a vital link between ATP and ROS production [247]. UCP2 protects against mitochondrial oxidative damage by reducing ROS [248]. UCP2 overexpression studies have confirmed its essential role in reducing oxidative stress, as ROS production was successfully decreased [249,250]. UCP2 is associated with DM [251]. UCP2 may be a promising therapeutic target for treating type 2 diabetes mellitus because it regulates insulin secretion and beta-cell dysfunction [252]. Since UCP2 plays a role in reducing ROS production, the selective activation of UCP2 may have therapeutic potential in patients with diabetes [253].

NF-κB, also known as nuclear factor-κB, is a nuclear transcription factor found in all cell types. ROS can activate NF-κB, which can modulate oxidative stress [254]. Studies performed in various cells and experimental animals have shown the role of NF-κB in the pathogenesis of diabetes mellitus. Increased NF-κB activation in the basal state and in response to TNF-α was observed at elevated glucose concentrations, indicating its potential relevance to diabetic complications [255]. Berberine can reduce NF-κB levels in the pancreas and liver of diabetic rats [203,204]. In other studies, the administration of berberine to diabetic animal models was reported to inhibit the activation of the NF-κB signaling pathway [256,257].

## 5. Antidiabetic Activity of Berberine

The effect of berberine on type 2 diabetes mellitus was first reported by Chen and Xie in 1986. Berberine, the main component of *Coptis chinensis*, showed a hypoglycemic effect in diabetic test animals [258]. In 1988, berberine was reported to have a hypoglycemic effect when used to treat diarrhea in patients with diabetes in China [259]. Since then, berberine has often been used as an antihyperglycemic agent in China. Several clinical trials related to the hypoglycemic effect of berberine have been reported in the literature in China.

Yin et al. compared the effects of berberine and metformin. In a three-month trial, 36 patients with type 2 diabetes mellitus were randomly assigned to berberine or metformin intervention groups. Berberine exhibited a hypoglycemic effect comparable to metformin, as indicated by decreased HbA1c, fasting blood glucose, and postprandial blood glucose. The total cholesterol and LDL cholesterol also significantly decreased. According to these findings, berberine may have potential as an oral hypoglycemic agent and is known to exert beneficial effects on lipid metabolism [260]. A similar clinical experiment by Zhang et al. showed that berberine dramatically lowered the fasting blood glucose, HbA1c, triglyceride, and insulin levels in patients with type 2 diabetes. Berberine can lower fasting blood sugar and HbA1c, similar to metformin and rosiglitazone [261].

A meta-analysis of 27 clinical trials by Lan et al. revealed that berberine has a therapeutic effect on type 2 diabetes mellitus, hyperlipidemia, and hypertension compared with other therapeutic regimens [87]. In another meta-analysis by Wei et al., seventeen studies involving 1198 patients were analyzed. Berberine significantly improved fasting blood glucose, postprandial blood glucose, HbA1c, and insulin resistance. Berberine showed a beneficial effect on the control of blood glucose levels. Although not better than metformin, berberine showed a better effect on fasting blood glucose levels than rosiglitazone. The combination of berberine with other oral hypoglycemic drugs is a new alternative treatment for diabetes mellitus [262]. Liang et al. performed a meta-analysis of 28 studies related to berberine clinical trials involving 2313 patients with type 2 diabetes mellitus. Berberine administered alone or in combination with oral hypoglycemic drugs improved fasting blood glucose, postprandial blood glucose, and HbA1c in patients with type 2 diabetes mellitus. However, berberine combined with oral hypoglycemic drugs showed a better reduction in fasting blood glucose and postprandial blood glucose levels than berberine or oral hypoglycemic drugs alone [12].

In addition to type 2 diabetes mellitus, several studies on berberine activity in gestational diabetes mellitus have also been conducted. It is known that all levels of glucose intolerance during pregnancy or first detected during pregnancy are referred to as gestational diabetes mellitus; however, this type of diabetes is usually diagnosed in the second or third trimester of pregnancy. This definition does not rule out the possibility that glucose intolerance exists before pregnancy or starts with pregnancy [1]. Gestational diabetes mellitus usually appears as a temporary disorder during pregnancy and resolves after pregnancy. Women with gestational diabetes mellitus are at greater risk of adverse pregnancy outcomes. These include high blood pressure (including pre-eclampsia) and a large baby for gestational age (macrosomia), which can make a normal birth difficult and hazardous, with the baby more prone to fractures and nerve damage. Babies born to mothers with gestational diabetes mellitus also have a higher lifetime risk of obesity and type 2 diabetes mellitus. Gestational diabetes mellitus is caused by relative insulin resistance and deficiency that accompanies pregnancy and occurs in nearly 3–5% of pregnant women. In 2021, 21.1 million (16.7%) live births to women involved some form of hyperglycemia during pregnancy. Of these, 80.3% were caused by gestational diabetes mellitus, 10.6% were caused by diabetes detected before pregnancy, and 9.1% were caused by diabetes (including type 1 and type 2) that was first detected in pregnancy [2].

Berberine is reported to improve insulin resistance conditions and maternal–fetal outcomes of gestational diabetes mellitus rats, such as the number of dead and absorbed fetuses, maternal body weight gain, and fetal and placental weight [263]. Maternal obesity and diabetes during pregnancy predispose to the development of cardiometabolic diseases. Berberine treatments protected against obesity, increased glucose-stimulated insulin secretion from pancreatic islets, and improved cardiac dysfunction [264]. Berberine protects against cardiac dysfunction in gestational diabetes mellitus, involving improved mitochondrial function mediated through increased cardiolipin synthesis [265]. Cardiolipin is a key mitochondrial membrane phospholipid that regulates cardiac mitochondrial bioenergetics [266].

Many studies on berberine’s mechanism as an antidiabetic have been conducted. Berberine is reported to act by several mechanisms, including increasing insulin secretion, improving insulin resistance, inhibiting gluconeogenesis, increasing glucose uptake, inducing glycolysis, inhibiting the action of several important enzymes, and regulating the gut microbiota [77,267].

### 5.1. Berberine Increases Insulin Secretion

Several in vitro and in vivo studies have shown that berberine increases insulin secretion. Lang et al. conducted a study on the effect of berberine in experimental animals with impaired glucose tolerance and its effect on insulin secretion in HIT-T15 cells and pancreatic islets. HIT-T15 is a cell culture sensitive to glucose and plays a role in secreting insulin. Berberine concentrations of 1–10 mol/L increased insulin secretion in HIT-T15 and pancreatic islet cell cultures [268]. Another study by Zhou et al. showed that berberine could increase insulin secretion and expression, and regenerate pancreatic beta cells in experimental rats with diabetes mellitus [171].

HNF-4α, a steroid hormone receptor superfamily member, is expressed in the liver, kidneys, small intestine, and pancreas (including pancreatic beta cells). HNF-4α directly activates the insulin gene. In addition, HNF-4α plays an important role in regulating and maintaining normal insulin secretion from pancreatic beta cells. The decrease in HNF-4α levels causes pancreatic beta cell damage and GSIS disorders and is associated with the emergence of type 2 diabetes mellitus [269]. Berberine increased glucose-stimulated insulin secretion (GSIS) in isolated experimental animal islets. The insulinotropic effects of berberine are mediated by increased nuclear factor 4-alpha (HNF-4α) expression and glucokinase (GK) activity. These two factors are not known to be involved in sulfonylurea-induced insulin secretion. Therefore, berberine is a promising insulin secretagogue that acts via a mechanism different from that of sulfonylureas [270].

Berberine also increased insulin secretion and glucokinase (GK) activity in NIT-1 cells. Berberine acts as a GK activator that increases glucose utilization, increases GK protein activity and expression, and decreases glucokinase regulatory protein (GKRP), an endogenous GK inhibitor [271]. GK activation is an alternative approach to improve glycemic control in patients with type 2 diabetes mellitus. Studies have shown low GK activity in patients with type 2 diabetes mellitus. GK acts as a “glucose sensor” or “glucose receptor” in pancreatic beta cells. The activation of GK triggers insulin secretion, increases glucose uptake in the liver, and plays a role in the synthesis and storage of glycogen in the liver, thereby reducing hepatic glucose expenditure. When GK is activated, glucose (a substrate of GK) is converted into glucose-6-phosphate (G6P). G6P can activate glycogen synthase and act as a substrate for glycogen synthesis [272].

Lu et al. studied the mechanism of action of berberine as an antidiabetic agent. Berberine increases insulin secretion by increasing GLP-1 secretion [273]. Similar findings have been reported by Zhang et al. Berberine was shown to reduce blood glucose levels through the mitogen-activated protein kinase (MAPK) pathway and increase GLP-1 secretion in the intestine [274]. GLP-1 is an incretin hormone in addition to the glucose-dependent insulinotropic peptide (GIP) [275]. The incretin hormone is a polypeptide of the glucagon superfamily synthesized in the small intestine and regulates glucose levels by stimulating insulin secretion in response to the presence of food [276,277].

However, a clinical trial by Pérez-Rubio showed different results from previous studies. Berberine treatment caused a decrease in insulin secretion, as indicated by the insulinogenic index value decreasing from 0.78 ± 0.69 (baseline) to 0.62 ± 0.46 (three months). On the other hand, berberine was also reported to be able to increase insulin sensitivity, as indicated by the Matsuda index value, which increased from 2.10 ± 1.00 (baseline) to 3.10 ± 1.60 (three months). This result suggests that the ability of berberine to improve metabolic control is not through an increase in insulin secretion but through an increase in insulin sensitivity [278]. These findings are similar to those from Bai et al. Berberine inhibited glucose oxidation and ATP synthesis in the pancreatic islets of experimental animals by inhibiting mitochondrial respiration. The inhibition of ATP synthesis alters the ATP/ADP ratio, causes AMPK activation, and decreases insulin secretion from pancreatic beta cells via a K_ATP_-dependent pathway [279].

The role of AMPK in insulin secretion is still under debate [280]. Several studies have shown that AMPK activation inhibits glucose-induced insulin secretion [281,282,283,284]. However, other studies have shown the opposite, where AMPK activation increased insulin secretion [285,286,287].

According to Zhao et al., the ability of berberine to increase insulin secretion is related to the KCNH6 channel. Berberine is reported to be able to bind to the KCNH6 channel and close the KCNH6 channel. KCNH6 channel closure prolongs the depolarization of high-glucose-dependent cell membranes, thereby increasing insulin secretion [288]. As mentioned earlier, insulin secretion from pancreatic beta cells is mediated by K_ATP_ channels, which cause membrane depolarization. However, in addition to the K_ATP_ channel, another potassium channel also plays a role in insulin secretion in humans and animals, namely, KCNH6 (non-K_ATP_ K^+^ channel). KCNH6 is a voltage-dependent K^+^ (Kv) channel [289]. In vitro studies have demonstrated the effect of non-K_ATP_ K^+^ channels on insulin secretion, particularly the voltage-dependent K^+^ (Kv) channel, which modulates insulin secretion [290,291]. Glucose-induced insulin secretion due to Kv channel closure has also been reported in experimental animal islets and insulinoma cells [292,293]. Other studies have also shown that glucose-induced insulin secretion from pancreatic beta cells is affected by high-glucose-dependent repolarization caused by Kv channels such as KCNH6 [294].

### 5.2. Berberine Improves Insulin Resistance

Several studies have been conducted on AMPK activation by berberine and its relationship with insulin resistance. AMPK plays an important role in regulating the energy homeostasis of cells and is activated by binding an ADP or AMP molecule to the regulatory site of the γ subunit [295]. AMPK plays a role in many metabolic processes, such as reducing energy stores and increasing energy production [296]. AMPK is known to be one of the potential therapeutic targets to improve insulin resistance [297].

Chang et al. conducted a study on the effects and mechanisms of berberine on glucose consumption and glucose uptake in insulin-resistant H9c2 cells. Berberine improves insulin resistance in H9c2 cells by increasing AMPK activity. Under conditions of insulin resistance, AMPK activation by berberine can increase GLUT4 translocation and glucose transport, thereby increasing glucose consumption and glucose uptake in insulin-resistant H9c2 cells [298]. In another study by Lee et al., berberine increased AMP-activated protein kinase (AMPK) activity in 3T3-L1 adipocytes and L6 myotubes, increased GLUT4 translocation in L6 cells, and decreased lipid accumulation in 3T3-L1 adipocytes. This result suggests that berberine has a beneficial effect on treating diabetes and obesity through the stimulation of AMPK activity [299]. Berberine can activate AMPK by inhibiting mitochondrial respiratory complex I and increasing insulin sensitivity in insulin-resistant experimental animals [300]. Mitochondrial complex I is one of the targets in treating diabetes mellitus. Drugs such as metformin and thiazolidinedione have also been reported to inhibit mitochondrial respiratory complex I [301].

Li et al. reported that berberine could increase insulin sensitivity and improve metabolic abnormalities in fructose-induced insulin resistance in experimental animals through activation of the LKB1/AMPK/PGC1α pathway. LKB1 is one of the kinases that play a role in AMPK activation, while PGC1α is involved in insulin-stimulated AKT phosphorylation. AKT is a key molecule in the insulin-signaling pathway that stimulates glycogen synthesis. PGC1α is known to increase AKT and p-AKT expression in muscles. PGC1α is an important transcriptional coactivator that plays a role in stimulating mitochondrial biogenesis and regulating energy metabolism. In this study, there was a decrease in the expression of PGC1α in insulin-resistant experimental animals, which could be improved by administering berberine. This result suggests that the protective effect of berberine against insulin resistance may be related to the upregulation of PGC1α [302].

HIF-2α plays a crucial role in regulating ceramide synthesis. Several recent clinical trials have shown that ceramide is strongly associated with insulin resistance. The ratio of ceramide C18/16 has been identified as a biomarker to predict diabetes and is a risk factor for diabetes mellitus. Ceramide affects glucose metabolism in the liver, and increased ceramide levels can induce insulin resistance. The inhibition of ceramide synthase can reduce ceramide production, thereby improving insulin resistance and obesity [303,304,305]. In vivo studies have shown that berberine can inhibit the accumulation of HIF-2α through the downregulation of the HIF-2α target gene, thereby reducing ceramide production and improving insulin resistance induced by high-fat diets in experimental animals [306].

Under hyperglycemic conditions, toxic compounds are produced from glucose, including methylglyoxal (MGO). MGO influences the pathogenesis of microvascular and macrovascular complications in diabetes mellitus. MGO causes vascular damage to endothelial cells and plays a role in developing insulin resistance, hypertension, diabetic neuropathy, and nephropathy. An increase in MGO levels has been reported in patients with type 2 diabetes mellitus [307]. Memon et al. conducted a clinical trial on the effect of berberine on methylglyoxal (MGO) levels and insulin resistance in patients with newly diagnosed type 2 diabetes mellitus compared with metformin. Metformin and berberine reduced the MGO levels by 43% and 56%, respectively. Likewise, the HOMA-IR value for berberine decreased by 29.50% compared to metformin, which was only 8.12%. This result suggests that berberine is more effective in lowering MGO levels and improving insulin resistance by improving glycemic control in newly diagnosed type 2 diabetes mellitus [308]. Similarly, berberine increased insulin sensitivity in obese experimental animals, reduced fasting insulin levels by 46%, and reduced the homeostatic model assessment of insulin resistance index (HOMA-IR) by 48% [309].

Kong et al. studied the molecular mechanism by which berberine improves insulin resistance. In this study, the insulin receptor was used as a target of berberine to increase insulin sensitivity. The results showed that berberine improved insulin resistance by increasing insulin receptor expression by activating protein kinase C (PKC). In vivo tests on experimental animals with type 2 diabetes mellitus showed that berberine reduced fasting blood glucose levels and fasting serum insulin levels, increased insulin sensitivity, increased the amount of insulin receptor mRNA, and increased PKC activity in the liver [310]. Similar findings were also reported by Gu et al., who reported that berberine prevented insulin resistance by regulating the expression of insulin receptors, insulin receptor substrate-1 (IRS-1), and glucagon in insulin-resistant experimental animals induced by a high-fat diet [311].

An increase in circulating branched-chain amino acids is implicated in the pathogenesis of obesity and insulin resistance. Berberine can reduce insulin resistance by increasing branched-chain amino acid catabolism in peripheral tissues and reducing branched-chain amino acid-producing bacteria, including the order *Clostridiales*, families *Streptococcaceae*, *Clostridiaceae*, and *Prevotellaceae*, and genera *Streptococcus* and *Prevotella* [312]. In another study, the effects of berberine on insulin-induced signal transduction and glucose uptake in insulin-sensitive and insulin-resistant rat skeletal muscle cells were observed. Berberine increases insulin-induced tyrosine phosphorylation of insulin receptors. Under insulin resistance conditions, berberine exhibited a synergistic effect on insulin-induced glucose uptake and GLUT4 translocation. This result suggests that berberine can overcome insulin resistance through the modulation of key molecules in insulin signaling pathways, resulting in increased glucose uptake in insulin-resistant cells [313].

Protein phosphatase Mg^2+^/Mn^2+^-dependent 1B (PPM1B) is a single-subunit enzyme that requires magnesium/manganese to function. PPM1B, also known as PP2Cβ, belongs to the Ser/Thr protein phosphatase (PP2C) family. Endogenous PPM1B is present in the nucleus of mature 3T3-L1 adipocytes and can bind to the PPARγ receptor [314]. Berberine improves insulin resistance through mechanisms related to regulating the PPM1B signaling pathway, including the cAMP/PKA/PPM1B, PPM1B/GLUT4, PPM1B/IKKβ/NF-κB, and PPM1B/PI3K/AKT pathways. Therefore, the ability of berberine to protect experimental animals with type 2 diabetes mellitus from insulin resistance is due to the regulation of the expression of cAMP, PKA, PPM1B, PPARγ, LRP1, GLUT4, NF-B p65, JNK, IKKβ, IRS-1, IRS -2, PI3K, and AKT in the heart [315].

In women with gestational diabetes, berberine is known to reduce insulin resistance by inhibiting hypoxia-inducible factor-3α (HIF3A) methylation [316]. It has been reported that HIF3A methylation in women with gestational diabetes is higher than in normal pregnant women. Studies have shown a significant correlation between HIF3A methylation and insulin resistance in gestational diabetes mellitus [317].

### 5.3. Berberine Inhibits Gluconeogenesis

Hepatic glucose production is the sum of gluconeogenesis. Increased hepatic glucose production, specifically gluconeogenesis, leads to increased glucose release into the blood, which can cause hyperglycemia and subsequent diabetic organ damage [318]. In patients with type 2 diabetes, gluconeogenesis has been identified as the primary source of glucose production, while glycogenolysis was found not to contribute [319,320].

In a study by Xie et al., berberine decreased gluconeogenic genes in the liver [321]. The activity of transcription factors such as forkhead transcription factor O1 (FoxO1) was also decreased. FoxO1 is an important transcription factor in the control of gluconeogenesis in the liver. FoxO1 induces PEPCK and G6Pase gene transcription to initiate gluconeogenesis [322].

In cultured hepatocytes, berberine inhibited oxygen consumption and decreased intracellular ATP levels, thereby inhibiting gluconeogenesis and lipogenesis in the liver. Berberine can reduce fasting blood glucose levels by inhibiting gluconeogenesis in the liver independent of insulin action [321]. Berberine inhibits gluconeogenesis via the LKB1-AMPK-TORC2 signaling pathway [323,324].

AMPK is a heteromeric protein consisting of the α catalytic subunit and β and γ regulatory subunits that regulate glucose and lipid metabolism, including gluconeogenesis and lipogenesis processes in the liver. AMPK is strongly associated with gluconeogenesis and is activated by liver kinase B1 (LKB1), transforming growth factor-activated kinase 1 (TAK1), and Ca^2+^/calmodulin-dependent protein kinase (CaMKKβ). AMPK also plays a role in regulating glucose-6-phosphatase (G6Pase) and phosphoenolpyruvate carboxykinase (PEPCK), which affect gluconeogenesis to improve diabetes mellitus [325]. LKB1 is a serine/threonine kinase with a molecular weight of 50 kDa that plays a role in the phosphorylation and activation of the AMPK catalytic subunit at the T-loop residue of Thr^172^. In adult rat liver tissue, LKB1 regulates AMPK phosphorylation and controls the rate of gluconeogenesis [326]. TORC2 also plays an important role in gluconeogenesis. TORC2 is required for fasting hepatic gluconeogenesis. Under fasting conditions, there is an increase in glucagon released by the pancreas, which stimulates gluconeogenesis through the increased expression of several gluconeogenic genes through the cAMP-responsive CREB factor. Glucagon stimulates CREB activity by inducing the coactivator TORC2 [327,328].

Other studies have demonstrated that berberine suppresses gluconeogenesis in the liver by blocking the glucagon pathway. Berberine inhibits CREB phosphorylation in hepatocytes and the liver and causes the downregulation of gluconeogenic genes (PEPCK and G6Pase), leading to decreased hepatic glucose production [329]. Zhang et al. also reported that berberine inhibits gluconeogenesis via PEPCK1 [330]. PEPCK1 is an important enzyme in gluconeogenesis and plays a role in regulating glucose homeostasis [331].

### 5.4. Berberine Increases Glucose Uptake

Berberine can increase glucose uptake by inhibiting SIRT3 deacetylation [330]. Berberine also significantly increased the mitochondrial depolarization ratio and intracellular AMP levels via SIRT3 ubiquitination. The oxidation potential of berberine, which stimulates glucose uptake by activating the AMPK pathway, is promoted by SIRT3 protein breakdown via ubiquitination [332].

SIRT3 is a protein in the mitochondrial matrix that interacts with subunits in complex I mitochondrial respiration [332]. SIRT3 plays a role in regulating the function of complex I in the electron transport chain and maintaining basal ATP [333]. SIRT3 inhibition results in mitochondrial dysfunction and an increased AMP/ATP ratio, which activates AMPK [334]. The activation of the AMPK signaling pathway further enhances glucose uptake [332].

A different mechanism of berberine was demonstrated by Cok et al. In this study, the ability of berberine to induce glucose uptake in L929 fibroblast cells, a cell line that only expresses the GLUT1 transporter protein, was observed. Berberine increased glucose uptake by increasing the activity of GLUT1. However, the ability of berberine to increase glucose uptake in fibroblasts is not associated with AMPK activity [335].

Cheng et al. studied the effect of berberine on L6 myotubes in the skeletal muscles of experimental animals with diabetes mellitus. The L6 cell culture was used to investigate the insulin-stimulated glucose transport mechanism. Berberine has been reported to stimulate glucose uptake in L6 myotubes, and this effect is not via the insulin signaling pathway but via the AMP-AMPK-p38 MAPK pathway. In muscle cells, p38 mitogen-activated protein kinase (p38 MAPK) is strongly associated with GLUT4 [336].

Zhou et al. demonstrated that berberine increased glucose uptake in 3T3-L1 adipocytes in the absence of insulin. Berberine enhanced GLUT1-mediated glucose uptake in 3T3-L1 adipocytes by activating GLUT1 via the AMPK pathway. However, berberine did not significantly stimulate GLUT4 protein translocation. Berberine does not affect GLUT1 or GLUT4 protein gene expression [337]. In contrast, another study showed that berberine increased glucose transport activity in 3T3-L1 adipocytes by increasing the expression of GLUT1 without decreasing GLUT4 expression [338]. Berberine stimulated glucose uptake in 3T3-L1 adipocytes to the same extent as rosiglitazone [339].

### 5.5. Berberine Induces Glycolysis

Glycolysis has been associated with type 2 diabetes mellitus [340]. Glycolysis is one of the glucose metabolism pathways that regulate insulin secretion and the physiological functions of some tissues and organs. Therefore, it is essential to maintain glucose metabolism homeostasis in a cell-type-dependent manner. Under insulin deficiency or resistance conditions, glycolysis can become dysregulated, caused by improper quantities and activity of metabolic and regulatory enzymes. One of the goals of diabetes treatment is to increase the rate of glycolysis in important tissues and cells involved in regulating systemic glucose homeostasis by targeting key metabolic and regulatory enzymes [341].

Glycolysis is a process modulated by a set of enzymes and factors, including AMPK [342]. AMPK stimulates glycolysis by activating phosphofructokinase (PFK), an important enzyme that plays an important role in glycolysis [343,344].

Berberine can increase glucose metabolism by increasing glycolysis. The increase in glycolysis results from the inhibition of mitochondrial respiratory chain complex I, which causes the suppression of ATP synthesis, thereby inhibiting glucose oxidation in the mitochondria, where this effect is independent of AMPK activation. Increased glycolysis is characterized by increased glucose consumption in HepG2 hepatocytes and C2C12 myotube cells [345]. In vitro studies have shown that berberine can increase glucose metabolism, as indicated by increased glucose consumption in HepG2 hepatocytes that are not insulin-dependent. This action is similar to metformin. HepG2 cells were used to observe the glucose-lowering effect on hepatocytes through glucose consumption [346]. Ren et al. reported a similar finding. Berberine was able to increase glucose metabolism through the activation of AMPKα1 in HepG2 cells [347].

### 5.6. Berberine Inhibits Enzyme Activity

Two important enzymes are involved in the carbohydrate digestion process: α-amylase and α-glucosidase [348]. The inhibition of these two enzymes causes a delay in the digestion of carbohydrates in the digestive tract, slows glucose absorption into the blood, and causes a decrease in blood glucose levels. Therefore, inhibiting α-amylase and α-glucosidase is a strategy or therapeutic approach for patients with type 2 diabetes mellitus [349,350].

Berberine may exert an antihyperglycemic action in the digestive tract prior to absorption because of its poor gut wall absorption and low bioavailability. It is similar to acarbose, an antihyperglycemic drug with low bioavailability due to poor absorption, and its therapeutic activity is within the gastrointestinal tract [351].

Several studies have shown that berberine inhibits α-amylase and α-glucosidase activities. A study conducted by Zhao et al. showed that berberine has strong inhibitory activity against both α-amylase and α-glucosidase in vitro, with IC_50_ values of 50.83 µg/mL and 198.40 µg/mL, respectively. The inhibition type in α-amylase is non-competitive, whereas, in α-glucosidase, competitive inhibition is observed [352]. In Caco-2 cells, berberine reduces glucose absorption in the gastrointestinal tract by inhibiting α-glucosidase activity [353]. The inhibition of α-glucosidase by berberine was still lower than that by acarbose [354].

An in vivo study by Liu et al. reported that berberine administration significantly reduced the activity of disaccharide enzymes (lactase, maltase, and sucrase) and β-glucuronidase in the small intestine of streptozotocin-induced diabetic rats. In addition, the activity of this disaccharide enzyme still decreased 24 h after the last administration of berberine [355]. The half-life of berberine after oral administration is approximately six hours [356]. This result suggests that the long-term administration of berberine may reduce the expression of disaccharide enzyme genes [355].

In patients with type 2 diabetes mellitus, decreased levels of glucagon-like peptide-1 (GLP-1) and glucose-dependent insulinotropic polypeptide (GIP) have been reported to cause hyperglycemia [357]. GLP-1 and GIP are incretin hormones synthesized in the small intestine in response to food. These two hormones regulate blood glucose levels by stimulating insulin secretion from pancreatic beta cells (GLP-1 and GIP) and suppressing glucagon secretion from pancreatic alpha cells (GLP-1). However, both hormones have short half-lives (less than two minutes) due to rapid inactivation by the DPP-4 (dipeptidyl peptidase-4) enzyme. DPP-4 inhibitors play a role in inhibiting the degradation of GLP-1 and GIP [358]. DPP-4 inhibitors can control blood glucose levels by increasing insulin secretion, inhibiting glucagon secretion, and slowing gastric emptying [359]. DPP-4 inhibitors can also induce pancreatic beta-cell regeneration [360] and inhibit pancreatic beta-cell apoptosis [361,362]. Al Masri et al. reported that the human recombinant DPP-4 enzyme was inhibited by berberine with an IC_50_ of 13.30 μM, where this effect is close to hypoglycemic activity [363].

### 5.7. Berberine Regulates Gut Microbiota

The gut microbiota plays a vital role in metabolic diseases, including diabetes. Currently, the gut microbiota is a new research area in diabetes management. Studies in animals and humans have shown differences in the gut microbiota composition in patients with diabetes. Disturbances in the gut microbiota can directly or indirectly cause insulin resistance and type 2 diabetes mellitus [364]. In a clinical trial by Zhang et al., the administration of berberine (either alone or in combination with probiotics) significantly changed the gut microbiota composition. Approximately 78 species were induced by berberine treatment. Two of these were *Bacteroides* spp. and the taxa *Proteobacteria* induced by metformin treatment. Further metagenomic and metabolomic studies revealed that the hypoglycemic effect of berberine was mediated by the inhibition of deoxycholic acid biotransformation by *Ruminococcus bromii* [365]. Several taxa have been identified as antidiabetic bacteria. *Ruminococcus* strains have been reported to regulate bile acid metabolism [366,367]. The bile acid transformation pathway by microbes has become one of the targets of action of several antidiabetic agents, either to decrease 7α/β dehydroxylation or to alter bile salt deconjugation, leading to the modulation of host bile acids and ultimately mediating the hypoglycemic effect of drugs [368].

The normal human gut microbiota consists of two main phyla, *Bacteroidetes* and *Firmicutes*, which can degrade many complex glycans and increase energy metabolism. *Proteobacteria* have been reported to be a common factor associated with human diseases, whereas *Lactobacillaceae* are effective in treating metabolic disorders. An in vivo study by Yao et al. reported that the gut microbiota community richness and diversity were significantly increased by berberine administration. The berberine intervention group showed an increase in *Bacteroidetes* and *Lactobacillaceae* and a decrease in *Proteobacteria* and *Verrucomicrobia*. In addition, berberine administration reduces the concentration of aromatic amino acids in the large intestine and blood. High concentrations of aromatic amino acids in the blood are strongly associated with the risk of developing type 2 diabetes mellitus [369]. Similar findings by Zhao et al. showed that *Firmicutes*, *Bacteroidetes*, and the *Bacteroidetes*/*Firmicutes* ratio were modified after the oral administration of berberine [370].

In another study by Tian et al., the administration of berberine affected the gut microbiota composition by decreasing *Clostridium* clusters XIVa and IV and bile salt hydrolase (BSH) activity, which caused the accumulation of taurocholic acid (TCA). TCA accumulation is associated with the activation of Farnesoid X receptors in the gut, which can mediate bile salt, lipid, and glucose metabolism [371]. Berberine fumarate (BF) exerts a hypoglycemic effect by regulating the gut microbiota. BF increased the populations of *Bacteroidetes*, *Clostridium*, *Lactobacillales*, *Prevotellaceae*, and *Alloprevotella* and decreased *Bacteroidales*, *Lachnospiraceae*, *Rikenellaceae*, and *Desulfovibrio* [372]. On the other hand, berberine chloride can affect the composition of the gut microbiota, increasing the number of bacteria that produce short-chain fatty acids (e.g., *Butyricimonas*, *Coprococcus*, and *Ruminococcus*), reducing the number of pathogenic bacteria (e.g., *Prevotella* and *Proteus*), and increasing some probiotics (e.g., *Lactobacillus* and *Akkermansia*) [373].

## 6. Conclusions

The use of natural products as complementary or alternative medicines is gaining worldwide popularity. Berberine is an isoquinoline alkaloid with several pharmacological activities. In silico studies have shown that berberine has the potential to treat many diseases. As a natural compound, berberine is present in several medicinal plants. One of these is *Fibraurea tinctoria*, which has a long history of use in traditional medicine in Borneo to treat diabetes. Due to its antioxidant properties, berberine has attracted considerable attention. These qualities may help explain its effectiveness against diabetes mellitus through several biochemical pathways. Therefore, extensive studies on the potency of berberine-containing plants with demonstrated pharmacological activity should involve additional in vitro and in vivo studies.

## Figures and Tables

**Table 1:** Berberine and its metabolites.

No.	Berberine Metabolites	No.	Berberine Metabolites
1	Berberine	7	Palmatine
2	Berberrubine	8	3,9-Demethylpalmatine
3	Thalifendine	9	3,10-Demethylpalmatine
4	Demethyleneberberine	10	2,3,9-Trihydroxiberberine
5	Jathorrhizine	11	2,3,10-Trihydroxiberberine
6	Columbamine	12	Berberrubine-9-*O*-β-D-glucuronide
13	Thalifendine-10-*O*-β-D-glucuronide	20	3,10-Demethylpalmatine-10-*O*-sulfate
14	Demethyleneberberine-2,3-di-*O*-β-D-glucuronide	21	2,3,10-Trihydroxyberberine-2-*O*-sulfate
15	Jatrorrhizine-3-*O*-β-D-glucuronide	22	Glucuronide of demethyleneberberine
16	Columbamine-3-*O*-β-D-glucuronide	23	Glucuronide of 3,9-demethylpalmatine
17	Demethyleneberberine-2-*O*-sulfate	24	Hydroxylated berberine
18	Thalifendine-10-*O*-sulfate	25	Dihydroberberine
19	Jatrorrhizine-3-*O*-sulfate		

**Table 2:** In silico studies of berberine in several diseases and the protein targets.

Diseases	Protein Targets	References
Diabetes mellitus	Insulin receptor (IR)	[116,117]
	α-Amylase	[118,119]
	α-Glucosidase	[117,119,120]
	Dipeptidyl peptidase IV (DPP-IV)	[117,121]
	Glycogen phosphorylase	[122]
	Glucose transporter type 4 (GLUT-4)	[116]
	Phosphoinositide 3-kinase (PI3K), protein kinase B (Akt), glycogen synthase kinase 3 beta (GSK-3β), and Kelch-like ECH-associated protein-1 (Keap-1)	[123]
	Cytokine signaling 3 (Socs3), Cholesteryl Ester Transfer Protein (CETP), C-Jun N-terminal kinases-1 (JNK1), lamin A/C, Peroxisome Proliferator-Activated Receptor γ (PPAR-γ), adiponectin, aldose reductase	[119]
Cancer	BRAF and CRAF kinases	[124]
	AKT	[125,126]
	Epidermal growth factor receptor (EGFR), p38 mitogen-activated protein kinases (p38 MAPK), Extracellular-Regulated Kinase (ERK1/2)	[126]
	Human proteome	[127]
	Survivin	[128]
	Retinoid X Receptor (RXRα)	[129]
Alzheimer’s	Amyloid beta (Aβ) peptide	[130,131,132]
	Beta-secretase 1 (BACE1)	[131]
	Cyclooxygenase-2 (COX-2), TNF Alpha Converting Enzyme (TACE)	[133]
	Acetylcholinesterase (AChE)	[133,134,135]
	Butyrylcholinesterase (BuChE)	[135]
Parkinson’s	Phosphodiesterase (PDE4 and PDE10), α-synuclein, monoamine oxidase (MAO-B), Adenosine A_2A_ receptors (A2Ar)	[136]
Hyperlipidemia	Proprotein Convertase Subtilisin/Kexin type 9 (PCSK9), HMG-CoA reductase (HMGCR)	[137]
	Niemann Pick C1 Like-1 (NPC1L1), Lanosterol 14α-Demethylase (LDM), Squalene Synthase (SqS)	[138]
Hyperuricemia	Urate transporter 1 (URAT1)	[106]
Anti-inflammatory	5-Lipoxygenase (5-LOX), cyclooxygenase-2 (COX-2)	[139]
	Epidermal growth factor receptor erbB1 (EGFR)	[140]
	Glucocorticoid Receptor (GR)	[141]
Hepatoprotective	p38 mitogen-activated protein kinases (p38 MAPK), Nuclear factor kappa-B (NF-κB), and Kelch-like ECH-associated protein 1 (Keap-1)	[142]
Dermatitis	Toll-like receptors (TLR1-TLR2 heterodimer)	[143]
Leishmanial	N-Myristoyltransferase (NMT), Methionyl-tRNA synthetase (MetRS), Pteridine reductase 1 (PTR1), Oligopeptidase B (OPB)	[144]
Zika Virus	ZIKV NS2B-NS3 protease	[145]
Antibacterial	Bacterial efflux pump proteins and biofilm proteins of bacteria of *Bacillus subtilis*, *Escherichia coli*, *Pseudomonas aeruginosa*, *Staphylococcus aureus*	[146]
	The inner membrane transporter (MexY)	[147,148]
	The filamentous temperature-sensitive Z protein (FtsZ)	[149,150,151]
Antifungal	Mucormycosis Proteins	[152]
Anticholinesterase	R. microplus acetylcholinesterase (RmAChE 1)	[153]
Anti-Influenza	Neuraminidase (NA)	[154,155]
	Neuraminidase (NA) of different subtypes of influenza A: A/H1N1/1918, A/H1N1/2009pdm, H3N2/2010 wild type, H3N2/2010 D151G mutant, H5N1 wild type, and H5N1 H274Y mutant	[156]
Immunomodulatory	Tumor necrosis factor-α (TNF-α), Interleukin (IL-1β and IL-6), cyclooxygenase-2 (COX-2)	[157]
COVID-19	ACE2 receptor	[158,159,160]
	COVID-19 main protease (M^pro^ or 3CL^pro^)	[160,161,162,163,164,165,166]
	Spike glycoprotein of SARS-CoV-2	[159,160]
	SARS spike glycoprotein–Human ACE2 complex	[167]
	RNA-Dependent RNA Polymerase of SARS-CoV-2	[166]
	Non-structural protein 15 (Nsp15)	[168]
	NFκB1, CHUK, MAPK3, MAPK1, NFκB1A, CASP3, IL6, MAPK8, BAX, TNF, TMPRSS2, PLpro, RdRp	[160]

**Table 3:** Effect of berberine on antioxidant parameters in diabetic animal models.

Animal Test	Diabetes Inducer	Berberine Dose (mg/kg/day)	Treatment Duration (Weeks)	Specimen	Findings	Ref.
Wistar rats	STZ 60 mg/kg, single i.p. injection High glucose	200 (Berberine)	12	Serum	-	[190]
				Cultured rat mesangial cells (in vitro)	MDA *, SOD **	
SD rats	STZ, 60 mg/kg, single tail-vein injection	200 (Berberine)	12	Serum	MDA *, SOD **	[191]
Wistar rats	STZ 35 mg/kg, single i.p. injection and HFD	75, 150, 300 (Berberine)	16	Serum	MDA *, GSH **, SOD **, GSH-Px **, CAT **	[192]
				Liver	MDA *, GSH **, SOD **, GSH-Px **, CAT **	
Mice	STZ 100 mg/kg, single i.p. injection	200 (Berberine chloride)	2	Liver	GSH *, SOD **, GSH-Px *	[188]
ICR Mice	STZ 100 mg/kg, single i.p. injection and Nicotinamide 1000 mg/kg, single i.p. injection	100 (Berberine chloride)	2	Liver	MDA *, SOD ***, CAT **	[189]
				Brain	MDA *, SOD **, CAT **	
				Kidney	MDA ***, SOD **, CAT *	
SD rats	HFD	100, 200 (Berberine chloride)	8	Kidney	MDA *, SOD **	[193]
Wistar rats	STZ 35 mg/kg, single i.p. injection and HFD	75, 150, 300 (Berberine chloride)	16	Pancreas	MDA *, SOD **	[171]
Wistar rats	Alloxan 55 mg/kg, single tail-vein injection and HFD	100, 200 (*Rhizoma coptidis*)	3	Heart	MDA *, SOD **, GSH-Px **	[194]
Wistar rats	STZ 60 mg/kg, single i.p. injection	25, 50, 100 (Berberine hydrochloride)	4	Cortex	MDA *, GSH **	[195]
				Hippocampus	MDA *, GSH **	
Albino Wistar rats	STZ 55 mg/kg, single i.p. injection	50, 100 (Berberine hydrochloride)	8	Hippocampus	MDA *, SOD **	[196]
Hamster	High glucose and HFD	50, 100 (Berberine chloride)	6	Serum	MDA *, TBARS * 8-isoprostane *, SOD **	[197]
SD rats	STZ 35 mg/kg, single i.p. injection and HFD	50, 100, 150 (Berberine chloride)	6	Liver	MDA **, SOD **, GSH **, GSSG ***, GSH:GSGG ***	[198]
Albino Wistar rats	Alloxan 100 mg/kg	250 (50% aqueous ethanolic root extract of *Berberis aristate*)	3	Liver	CAT **, SOD **, GR **, GSH **, GPx **, MDA *, Protein Carbonyl *	[199]
Wistar rats	STZ 60 mg/kg, single i.p. injection	200 (Berberine)	12	Serum	MDA *, SOD **	[200]
SD rats	STZ 80 mg/kg, single i.p. injection (twice)	5, 10, 20 (Berberine chloride)	2	Liver	MDA *, SOD *, CAT *, GPx *	[201]
ICR Mice	STZ 30 mg/kg, single i.p. injection	5 (Berberine)	3	Pancreas	MDA *, SOD **	[202]
Albino Wistar rats	STZ 40 mg/kg, intragastric intubation	50 (Berberine chloride)	7	Pancreas	SOD **, TBARS *, LOOH *, CAT **, GPx **, GSH **	[203]
Albino Wistar rats	STZ 40 mg/kg, intragastric intubation	50 (Berberine chloride)	7	Liver	SOD **, TBARS *, LOOH *, CAT **, GPx **, GSH **	[204]
Albino rats	STZ 35 mg/kg, single i.p. injection and HFD	50, 100 (Berberine chloride)	4	Liver	MDA *, SOD **, CAT **, GPx **, GSH **	[205]
Wistar rats	STZ 35 mg/kg, single i.p. injection and HFD	50, 100 (Berberine)	12	Serum	SOD **, CAT **, GPx **, GST **	[206]
Wistar rats	STZ 60 mg/kg, single i.p. injection	50 (Berberine chloride)	4	Lense	SOD ***, CAT ***, GPx ***, TBARS ***	[207]
Rats	STZ 50 mg/kg, single i.p. injection	50, 100 (Berberine)	2	Brain	MDA *, SOD **, GPx **, GSH **	[208]

*: Decrease; **: increase; ***: no effect; MDA: malondialdehyde; SOD: superoxide dismutase; GSH-Px/GPx: glutathione peroxidase; GSH: glutathione; GSSG: glutathione disulfide; CAT: catalase; GR: glutathione reductase; GST: glutathione transferase; LOOH: lipid hydroperoxides; TBARS: thiobarbituric acid-reactive substances; STZ: streptozotocin; i.p.: intraperitoneal; HFD: high-fat diet.

## Data Availability

The study did not report any data.
